# Supplementary material for: Changes in 25-(OH) Vitamin D Levels during the SARS-CoV-2 Outbreak: Lockdown-Related Effects and First-to-Second Wave Difference—An Observational Study from Northern Italy
Source: Biology (Basel). 2021 Mar 19;10(3):237. doi: 10.3390/biology10030237 (PMC8003430; doi:10.3390/biology10030237)
Supplement: Supplementary file 1 [file biology-10-00237-s001.zip › biology-1107744-supplementary.docx]

Article

Changes in 25-(OH) Vitamin D Levels during the SARS-CoV-2 Outbreak: Lockdown-Related Effects and First-to-Second Wave Difference—An Observational Study from Northern Italy

Davide Ferrari ^1^, Massimo Locatelli ^2^, Martina Faraldi ^3^ and Giovanni Lombardi ^3,4,^*

^1^ SCVSA Department, University of Parma, 43121 Parma, Italy; [davide.ferrari@unipr.it](mailto:davide.ferrari@unipr.it)

^2^ Laboratory Medicine Service, San Raffaele Hospital, 20132 Milano, Italy; [locatelli.massimo@hsr.it](mailto:locatelli.massimo@hsr.it)

^3^ Laboratory of Experimental Biochemistry & Molecular Biology, IRCCS Istituto Ortopedico Galeazzi,
20161 Milano, Italy; [martina.faraldi@grupposandonato.it](mailto:martina.faraldi@grupposandonato.it)

^4^ Department of Athletics, Strength and Conditioning, Poznań University of Physical Education,
61-871 Poznań, Poland

***** Correspondence: giovanni.lombardi@grupposandonato.it or [lombardi@awf.poznan.pl](mailto:lombardi@awf.poznan.pl);
Tel.: +39-02-6621-4068

**Scheme 1.** Averaged 25-(OH)D concentrations, SARS-CoV-2 cases and COVID-19-related deaths every million inhab-14 itants in nineteen European countries and Turkey (updated version from , last update January 9th, 2021).

| **Country** | **25-(OH)D (nmol/L)** | **SARS-CoV-2 cases/1M pop** | **COVID-19-related deaths/1M pop** |
| --- | --- | --- | --- |
| **Belgium** | 49.3 | 56878 | 1721 |
| **Czech Republic** | 62.5 | 76750 | 1211 |
| **Denmark** | 65 | 31060 | 266 |
| **Estonia** | 51 | 24935 | 209 |
| **Finland** | 67.7 | 6923 | 106 |
| **France** | 60 | 42038 | 1032 |
| **Germany** | 50.1 | 22606 | 482 |
| **Hungary** | 60.6 | 35290 | 2094 |
| **Ireland** | 56.4 | 27360 | 469 |
| **Iceland** | 57 | 17173 | 85 |
| **Italy** | 50 | 37042 | 1290 |
| **Netherlands** | 59.5 | 41085 | 1244 |
| **Norway** | 65 | 10008 | 87 |
| **Portugal** | 39 | 45841 | 746 |
| **Slovakia** | 81.5 | 37582 | 519 |
| **Spain** | 42.5 | 43845 | 1109 |
| **Sweden** | 73.5 | 48308 | 931 |
| **Switzerland** | 46 | 55018 | 949 |
| **Turkey** | 51.8 | 27208 | 265 |
| **UK** | 47.4 | 43446 | 1173 |
